# A novel liver-function-indicators-based prognosis signature for patients with hepatocellular carcinoma treated with anti-programmed cell death-1 therapy

**DOI:** 10.1007/s00262-024-03713-6

**Published:** 2024-06-04

**Authors:** Zehao Zheng, Jie Mei, Renguo Guan, Jiqi Zhang, Xinhao Xiong, Junyu Gan, Shaohua Li, Rongping Guo

**Affiliations:** 1 Department of Liver Surgery, Sun Yat-Sen University Cancer Center, Guangzhou, China; 2 State Key Laboratory of Oncology in South China, Collaborative Innovation Center for Cancer Medicine, Guangdong Provincial Clinical Research Center for Cancer, Sun Yat-Sen University Cancer Center, Guangzhou, China; 3 Sun Yat-Sen University, Guangzhou, China

**Keywords:** Liver function, Hepatocellular carcinoma, Anti-PD-1 therapy, Survival, APAR, PAL, ALRI, GLR

## Abstract

**Background:**

The liver function reserve has a significant impact on the therapeutic effects of anti-programmed cell death-1 (PD-1) for hepatocellular carcinoma (HCC). This study aimed to comprehensively evaluate the ability of liver-function-based indicators to predict prognosis and construct a novel prognostic score for HCC patients with anti-PD-1 immunotherapy.

**Methods:**

Between July 2018 and January 2020, patients diagnosed with HCC who received anti-PD-1 treatment were screened for inclusion in the study. The valuable prognostic liver-function-based indicators were selected using Cox proportional hazards regression analysis to build a novel liver-function-indicators-based signature (LFIS). Concordance index (C-index), the area under the receiver operating characteristic (ROC) curve (AUC), and Kaplan–Meier survival curves were utilized to access the predictive performance of LFIS.

**Results:**

A total of 434 HCC patients who received anti-PD-1 treatment were included in the study. The LFIS, based on alkaline phosphatase-to-albumin ratio index, Child–Pugh score, platelet-albumin score, aspartate aminotransferase-to-lymphocyte ratio index, and gamma-glutamyl transpeptidase-to-lymphocyte ratio index, was constructed and identified as an independent risk factor for patient survival. The C-index of LFIS for overall survival (OS) was 0.692, which was higher than the other single liver-function-based indicator. The AUC of LFIS at 6-, 12-, 18-, and 24-month were 0.74, 0.714, 0.747, and 0.865 for OS, respectively. Patients in the higher-risk LFIS group were associated with both worse OS and PFS. An online and easy-to-use calculator was further constructed for better application of the LFIS signature.

**Conclusion:**

The LFIS score had an excellent prognosis prediction ability superior to every single liver-function-based indicator for anti-PD-1 treatment in HCC patients. It is a reliable, easy-to-use tool to stratify risk for OS and PFS in HCC patients who received anti-PD-1 treatment.

**Supplementary Information:**

The online version contains supplementary material available at 10.1007/s00262-024-03713-6.

## Background

Hepatocellular carcinoma (HCC) is one of the most common causes of cancer-related death worldwide [1]. HCC patients are often diagnosed at an advanced stage with relatively poor five-year survival [2]. Recently, significant progress has been made in the systemic treatments of advanced HCC [3]. Immune checkpoint inhibitors (ICI), especially anti-programmed cell death-1 (PD-1) antibodies, as effective immunotherapeutic agents, effectively improve the survival of HCC [4]. However, the efficacy of anti-PD-1 antibodies varies greatly among advanced HCC patients. There are currently no validated biomarkers to identify the patients who could benefit from immunotherapy [5].

Most advanced HCC patients are accompanied by chronic liver disease [6]. Liver function has a significant impact on ICI therapeutic effects [7, 8]. The Child–Pugh score system evaluates the liver function of patients with chronic liver disease based on five clinical variables [9]. Studies have shown that HCC patients at the Child–Pugh A level prefer a better prognosis after surgical resection or systemic treatments [10]. In recent years, the proportion of liver function grade A in liver cancer patients has increased with the continuous improvement of hepatoprotective therapy, which leads to a weakened effect of the Child–Pugh system on prognosis. When liver parenchymal damage or decreased liver functional reserve occurs, the serum tests demonstrate increased levels of aspartate aminotransferase (AST), alanine aminotransferase (ALT), alkaline phosphatase (ALP), gamma-glutamyl transpeptidase (GGT), lactate dehydrogenase (LDH) and total bilirubin (TBIL) and a decreased level in albumin (ALB). Derived from these serum indicators, such as albumin-bilirubin index (ALBI) [9], platelet-albumin-bilirubin index (PALBI) [11], platelet-albumin index (PAL) [12], gamma-glutamyl transpeptidase-to-platelet ratio (GPR) [13], AST/ALT ratio (AAR) [14], fibrosis-4 (FIB-4) and aspartate-aminotransferase-to-neutrophil ratio index (ANRI) [15] are considered as the novel and non-invasive indicators for evaluating liver function or liver fibrosis of the patients with chronic liver diseases. Numerous studies have shown that these indicators can effectively evaluate the prognosis of HCC patients undergoing surgical resection [16–21]. Other blood biomarkers combined inflammation and liver function, including neo‐Glasgow prognostic score (neo-GPS) [22], aspartate aminotransferase-to-lymphocyte ratio index (ALRI) [23], gamma-glutamyl transpeptidase-to-lymphocyte ratio (GLR) [24], CRP-to-albumin ratio (CAR) [19]have also been reported as independent prognostic predictors for HCC. So far, there are limited studies on the prognostic values of these indicators for unresectable HCC patients receiving anti-PD-1 antibody treatments.

Therefore, the present study comprehensively evaluated the predictive ability of liver-function-based indicators for the determination of the prognosis and constructed a novel prognostic score and an online calculator for HCC patients who received anti-PD-1 inhibitors treatments.

## Materials and methods

### Study design and participants

From July 1, 2018 to January 1, 2020, patients who were diagnosed with advanced HCC and received anti-PD-1 inhibitors at Sun Yat-sen University Cancer Center (SYSUCC) were included in the present study. This study was approved by the ethics committee of SYSUCC (B2020-190-01). The inclusion criteria are as follows: (1) patients who were diagnosed with HCC by imaging or biopsy pathology according to American Association for the Study of Liver Diseases (AASLD) practice guidelines [25]; (2) aged between 18 and 80 years; (3) had received anti-PD-1 inhibitors; (4) had a performance status (PS) score ≤ 2; (5) liver functions at Child–Pugh A or B stage; (6) had no other malignant tumors; and (7) had complete medical and follow-up data.

### Anti-PD-1 Treatment

HCC patients received the anti-PD-1 inhibitors every 3–4 weeks until disease progression, unacceptable toxicity, or treatment abandonment. Anti-PD-1 inhibitors could be single used, or combined with locoregional therapies or other systemic treatments. The types and dosages of drugs are summarized in Supplementary Table 1.

The primary endpoint of the study was OS. The definition of liver-function-based indicators was listed in Supplementary Table 2. Similar to previous studies [26, 27], all peripheral blood test data were collected within 3 days before the initial administration of anti-PD-1 agents and at 8 weeks (PFS ≥ 8 weeks) or last blood test (PFS < 8 weeks) after the initial administration of anti-PD-1 agents. Enhanced computed tomography (CT) or magnetic resonance imaging (MRI) examination was used to evaluate the initial tumor situation and treatment response according to the modified RECIST (mRECIST) [28].

### Statistical analysis

Xtile v.3.6.1 software was used to identify the optimal cutoff value for the serum indicators [29]. Cox proportional hazards regression was used to evaluate the prognostic value of the serum indicators. The formula was used to calculate the LFIS score: Exp[Ʃ (βi (coefficient) * (the value of a variable)]. Schoenfeld residuals method was used in proportional hazards assumption test and the variables with *P*-value < 0.05 will be stratified in multivariate Cox regression. Similar to our previous study [20], tolerance and variance inflation factor (VIF) were applied to evaluate the multicollinearity and variables with tolerance < 0.1 and VIF > 10 were further excluded from the multivariate Cox regression. The concordance index (C-index) and area under the receiver operating characteristic (ROC) curve (AUC) were utilized to assess the discrimination indicators' performance. Survival analysis was performed using the Kaplan–Meier curve analysis. Groups were compared by using the Student’s t test for continuous data and the *χ*^2^ test for categorical data. R version 4.3.1 software (http://www.r-project.org/) and SPSS 26.0 were used for the statistical analyses. Github (http://github.com) and HTML5 (https://www.w3.org/TR/2008/WD-html5-20080122/) were used to build an online calculator. *P*-value < 0.05 was regarded as statistically significant and all tests were two-sided.

## Results

### Basic characteristics of patients

**Fig. 1:**
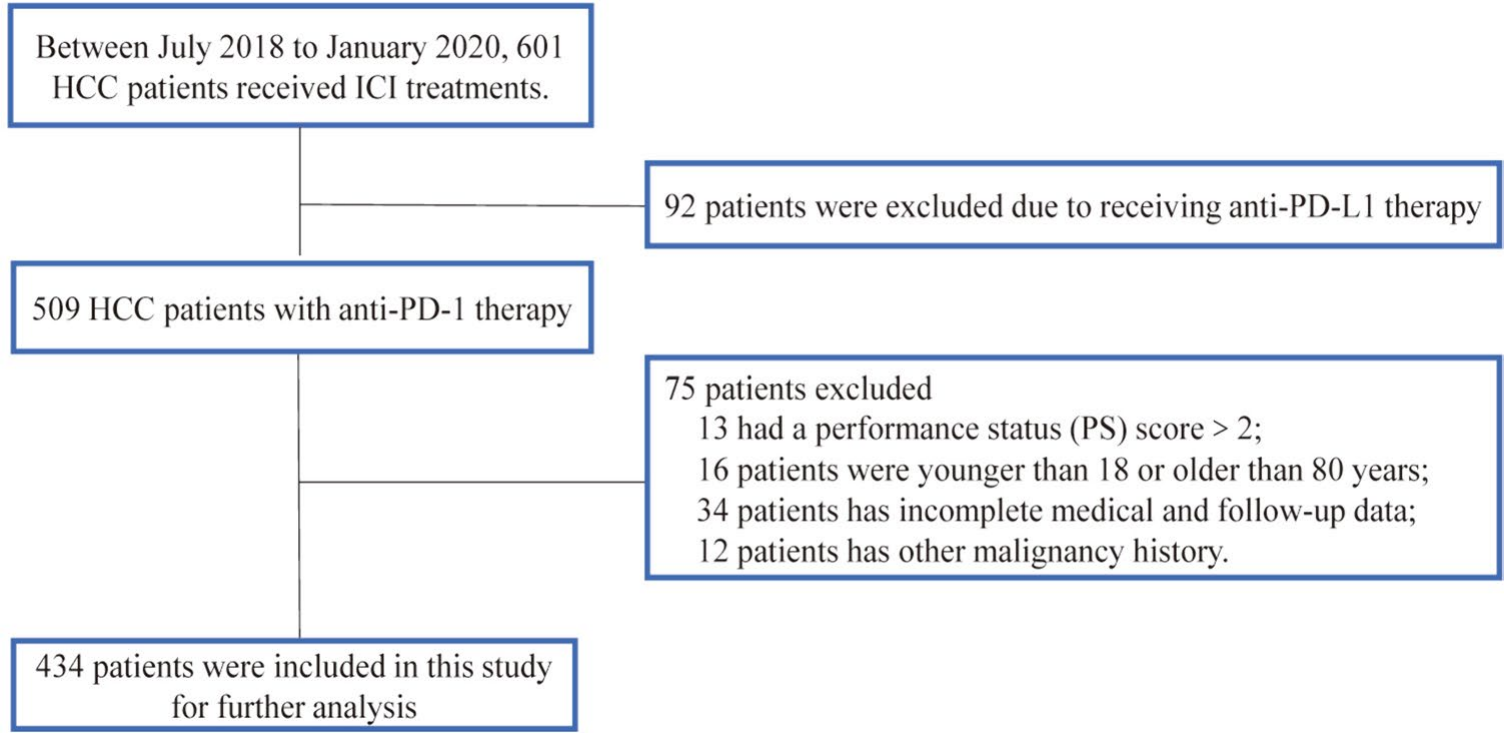
Flow chart of the study

Based on the inclusion criteria, 434 HCC patients who underwent anti-PD-1 inhibitors were eventually included (Fig. 1), among which 375 (86.41%) were males and 326 (75.12%) were older than 60 years. Approximately 71.66% of patients were diagnosed at Barcelona Clinic Liver Cancer (BCLC) stage C. Multiple tumors, macrovascular invasion, and extrahepatic metastasis accounted for 61.98, 52.53, and 39.17% of the patients, respectively. Before accepting the immunotherapy, 187 (43.09%) patients in this study received other antitumor treatments. As for the liver functional reserve, a total of 403 patients were with Child‐Pugh grade A, and only 31 (7.14%) patients were with Child–Pugh B before receiving anti-PD-1 inhibitors. Every single liver-function-based indicator got the best cutoff value based on the Xtile. The clinicopathological characteristics, including several liver function indicators, were summarized in Table 1.

**Table 1:** Baseline characteristics of the included patients

Characteristics	(*N* = 434)
*Gender*	
Female	59 (13.59%)
Male	375 (86.41%)
*Age*	
< 60	326 (75.12%)
≥ 60	108 (24.88%)
*Tumor number*	
0	29 (6.68%)
1	136 (31.34%)
Multiple	269 (61.98%)
*Tumor diameter*	
< 10	255 (58.76%)
≥ 10	179 (41.24%)
*Macrovascular invasion*	
No	206 (47.47%)
Yes	228 (52.53%)
*Extrahepatic metastasis*	
No	264 (60.83%)
Yes	170 (39.17%)
*BCLC*	
A–B	123 (28.34%)
C	311 (71.66%)
*Previous treatment*	
No	247 (56.91%)
Yes	187 (43.09%)
*Cycle of anti-PD1 therapy*	
< 4	200 (46.08%)
≥ 4	234 (53.92%)
*Hepatitis*	
No	62 (14.29%)
Yes	372 (85.71%)
*HBV-DNA copies*	
0	217 (50.00%)
< 2000	162 (37.33%)
≥ 2000	55 (12.67%)
*Combined therapy*	
No	48 (11.06%)
Yes	386 (88.94%)
*BR*	
NA	71 (16.36%)
CR	22 (5.07%)
PR	107 (24.65%)
SD	162 (37.33%)
PD	72 (16.59%)
*DCR*	
Response	291 (67.05%)
Non-response	72 (16.60%)
NA	71 (16.35%)
*Child–Pugh*	
A	403 (92.86%)
B	31 (7.14%)
*AFP*	
< 400	201 (46.31%)
≥ 400	233 (53.69%)
*PIVKA*	
< 40	35 (8.06%)
≥ 40	399 (91.94%)
*CRP*	
< 10	210 (48.39%)
≥ 10	224 (51.61%)
*PNI*	
< 48	212 (48.85%)
≥ 48	222 (51.15%)
*NLR*	
< 3.71	296 (68.20%)
≥ 3.71	138 (31.80%)
*ALB*	
< 35	65 (14.98%)
≥ 35	369 (85.02%)
*ALT*	
< 50	251 (57.83%)
≥ 50	183 (42.17%)
*AST*	
< 40	119 (27.42%)
≥ 40	315 (72.58%)
*ALP*	
< 108	183 (42.17%)
≥ 108	251 (57.83%)
*GGT*	
< 281.4	322 (74.19%)
≥ 281.4	112 (25.81%)
*LDH*	
< 259	271 (62.44%)
≥ 259	163 (37.56%)
*TBIL*	
< 20.5	319 (73.50%)
≥ 20.5	115 (26.50%)
*ALBI*	
1 (≤ − 2.6)	247 (56.91%)
2 ((> − 2.6 and ≤ − 1.39)	183 (42.17%)
3 (> − 1.39)	4 (0.92%)
*PALBI*	
1 (≤ -2.53)	170 (39.17%)
2 (> − 2.53 and ≤ − 2.09)	168 (38.71%)
3 (>− 2.09)	96 (22.12%)
*PAL*	
1 (≤ − 3.77)	268 (61.75%)
2 (> − 3.77 and ≤ − 3.04)	149 (34.33%)
3 (> − 3.04)	17 (3.92%)
*neoGPS*	
0	158 (36.41%)
1	141 (32.49%)
2	135 (31.11%)
*APAR*	
< 2.33	145 (33.41%)
≥ 2.33	289 (66.59%)
*GAR*	
< 5.73	297 (68.43%)
≥ 5.73	137 (31.57%)
*GPR*	
< 0.69	209 (48.16%)
≥ 0.69	225 (51.84%)
*FIB-4*	
< 88.15	369 (85.02%)
≥ 88.15	65 (14.98%)
*AAR*	
< 1.38	230 (53.00%)
≥ 1.38	204 (47.00%)
*APRI*	
< 2.46	388 (89.40%)
≥ 2.46	46 (10.60%)
*ALRI*	
< 100.15	357 (82.26%)
≥ 100.15	77 (17.74%)
*GLR*	
< 323.58	377 (86.87%)
≥ 323.58	57 (13.13%)
*CAR*	
< 0.13	147 (33.87%)
≥ 0.13	287 (66.13%)

BCLC, Barcelona Clinic Liver Cancer; HBV, Hepatitis B virus; BR, best response; NA, not assessed; CR, complete response; PR, partial response; SD, stable disease; PD, progressive disease; DCR, disease control rate; AFP, alpha fetoprotein; PIVKA-II, protein induced by vitamin K absence or antagonist-II; CRP, C-reactive protein; PNI, prognostic nutritional index; NLR, neutrophil to lymphocyte rate; ALB, albumin; ALT, alanine aminotransferase; AST, aspartate aminotransferase; ALP, alkaline phosphatase; GGT, gamma-glutamyl transpeptidase; LDH, lactate dehydrogenase; TBIL, total bilirubin; ALBI, albumin-bilirubin index; PALBI, platelet-albumin-bilirubin index; PAL, platelet-albumin index; neoGPS, neo-Glasgow prognostic score; APAR, alkaline phosphatase-to-albumin index; GAR, gamma-glutamyl transpeptidase-to- albumin ratio; GPR, gamma-glutamyl transpeptidase-to-platelet ratio;FIB-4, fibrosis-4; AAR, AST/ALT ratio; ALRI, aspartate aminotransferase-to-lymphocyte ratio index; GLR, gamma-glutamyl transpeptidase-to-lymphocyte ratio CAR, C-reactive protein to albumin ratio

The median follow-up duration was 17.1 months, while the median OS time was 18.3 months. 200 patients died and 320 patients experienced tumor progression during the follow‐up period. The tumor overall response rate (ORR) was 29.72%, while the disease control rate (DCR) was 67.05%.

## The correlations between tumor characteristics, anti-pd1 treatment and liver function

We performed the Chi-square test between tumor characteristics and liver function status. As Supplementary Table 3 shows, 12.30% of patients with tumors ≥ 10 cm had C-P B grade liver function and only 3.53% of patients with tumor < 10 cm had C-P B grade liver function. The patients with ALBI grade 2–3 had larger tumors and more macrovascular invasion.

After receiving anti-PD1 treatment, a total of 371 patients were available for liver function assessment. As Supplementary Fig. 1, 90.03% of HCC patients maintained initial Child–Pugh grade (A to A and B to B), while 27 patients had liver function deterioration (A to B). We further compared the changes in ALBI grade. 247 HCC patients maintained a consistent ALBI grade after treatment, while 21.29% of patients progressed from ALBI grade 1 to ALBI grade 2 or 3.

## Comparison of the prognostic prediction performance of the liver-function-based indicators

To further identify which liver-function-based indicators had better prognostic prediction performance, time-dependent ROC curves at 6-, 12-, 18-, and 24-month OS rates and the C-indexes were calculated. The APAR had a higher C-index value and better ability to predict OS than the other indicators (Table 2).

**Table 2:** Concordance Index for the Comparison of Different Functional liver reserve-based indicators

Indicators	C-Index	6-Month AUROC	12-Month AUROC	18-Month AUROC	24-Month AUROC
LFIS score	0.692	0.74	0.714	0.747	0.865
APAR	0.657	0.723	0.665	0.714	0.822
ALB	0.624	0.653	0.652	0.67	0.794
AST	0.624	0.702	0.628	0.634	0.765
TBIL	0.607	0.71	0.599	0.643	0.497
GGT	0.626	0.692	0.63	0.672	0.776
LDH	0.609	0.669	0.633	0.616	0.592
GAR	0.637	0.704	0.644	0.672	0.776
PAL	0.594	0.612	0.611	0.651	0.721
PALBI	0.626	0.665	0.647	0.638	0.703
neoGPS	0.618	0.664	0.65	0.624	0.715
FIB-4	0.614	0.662	0.641	0.591	0.692
AAR	0.616	0.675	0.65	0.611	0.568
APRI	0.58	0.65	0.572	0.622	0.661
GPR	0.597	0.659	0.592	0.668	0.67
GLR	0.639	0.705	0.654	0.676	0.763
ALRI	0.625	0.699	0.638	0.636	0.745
CAR	0.619	0.672	0.654	0.61	0.631
Child–Pugh	0.54	0.574	0.54	0.535	0.547

AUROC, under the receiver operating characteristic curve; LFIS, liver-function-indicators-based signature; ALB, albumin; ALT, alanine aminotransferase; AST, aspartate aminotransferase; ALP, alkaline phosphatase; GGT, gamma-glutamyl transpeptidase; LDH, lactate dehydrogenase; TBIL, total bilirubin; ALBI, albumin-bilirubin index; PALBI, platelet-albumin-bilirubin index; PAL, platelet-albumin index; neoGPS, neo-Glasgow prognostic score; APAR, alkaline phosphatase-to-albumin index; GAR, gamma-glutamyl transpeptidase-to- albumin ratio; GPR, gamma-glutamyl transpeptidase-to-platelet ratio;FIB-4, fibrosis-4; AAR, AST/ALT ratio; ALRI, aspartate aminotransferase-to-lymphocyte ratio index; GLR, gamma-glutamyl transpeptidase-to-lymphocyte ratio CAR, C-reactive protein to albumin ratio

**Fig. 2:**
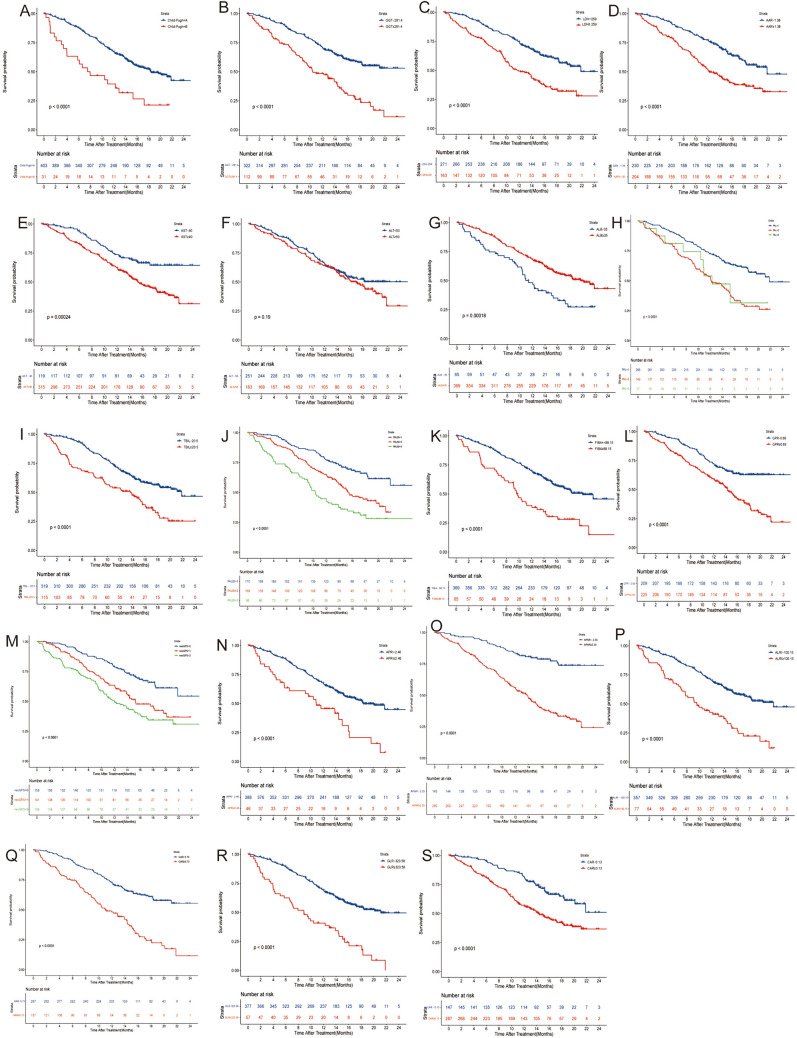
**A**–**S** Kaplan–Meier curves of the overall survival of HCC patients after anti-PD-1 treatment. **A** Child–Pugh, **B** GGT, **C** LDH, **D** AAR, **E** AST, **F** ALT, **G** ALB, **H** PAL, **I** TBIL, **J** PALBI, **K** FIB-4, **L** GPR, **M** neoGPS, **N** APRI, **O** APAR, **P** ALRI, **Q** GAR, **R** GLR, **S** CAR

The Kaplan–Meier survival analysis was conducted in all the indicators. As shown in Fig. 2, most of the liver-function-based indicators were significantly related to the OS of the HCC patients. Specifically, high Child–Pugh grade, APAR, GAR, GGT, TBIL, AST, ALBI, FIB4, AAR, GPR, GLR, CAR, ALRI, and LDH scores were associated with poorer prognosis (all *P* < 0.05), while high ALB levels were associated with better prognosis (*P* < 0.05).

## Construction and prognostic prediction performance of the novel liver-function-indicators-based signature

**Fig. 3:**
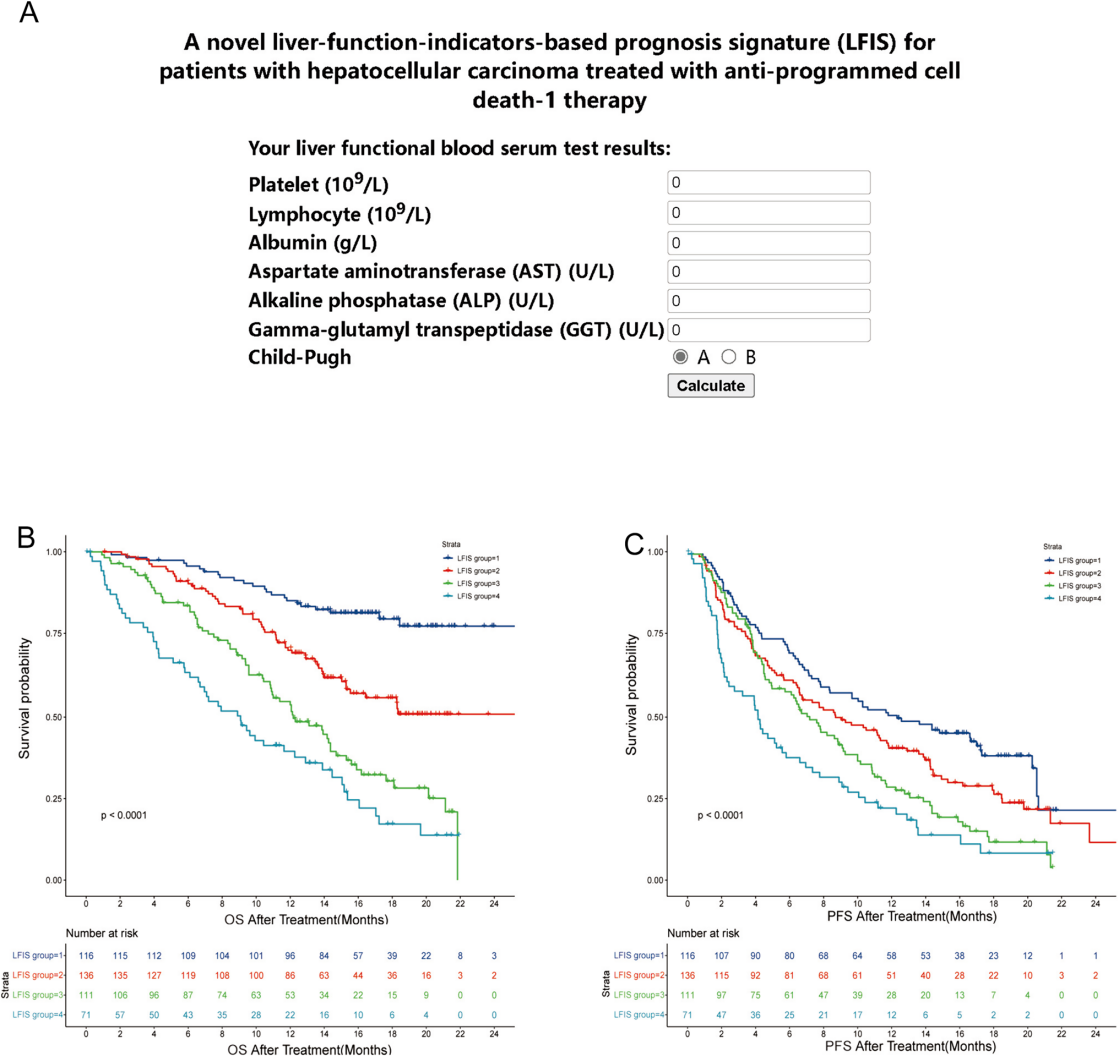
**A** The online calculator; **B** the OS curves according to the LFIS score. **C** The PFS curves according to the LFIS score. OS curves, *OS* overall survival, *PFS* progression-free survival

Univariate Cox analyses were performed to identify the liver function indicators associated with the OS of HCC patients. The liver-function-based indicators with *P* < 0.05 were further analyzed using stepwise multivariate COX regression. Finally, Child–Pugh score, PAL, APAR, ALRI, and GLR were identified as significant indicators for the OS of HCC patients who received anti-PD1-based treatments (Supplementary Table 4). LFIS was calculated based on the formula mentioned in Supplementary Table 5. The value of indicators was calculated by Xtile and coefficients of indicators were obtained by multivariate COX regression. The score of each indicator was acquired by multiplying their value and coefficient. Specifically, Child–Pugh A, PAL grade 1, APAR < 2.33, GLR < 323.58, and ALRI < 100.15 was assigned 0; Child–Pugh B was given 0.63319; PAL grade 1 was assigned 0.35054 and PAL grade 2 was given -0.10236; APAR ≥ 2.33 was given 0.98514; GLR ≥ 323.58 was assigned 0.58049; ALRI ≥ 100.15 was assigned 0.58714. Finally, the risk score, named “LFIS”, was calculated by taking an exponential function of the sum of each indicator score. To facilitate clinical application, we also have further designed an online calculator (Fig. 3A, https://ernie1.github.io/lfis_calculator/). The online calculator was compatible with browsing on a personal computer and mobile phone so that doctors or patients can visit our online calculator easily, and obtain LFIS risk score and group information by simply entering the results of pre-treatment examination indicators.

It had better predictive power than any other single liver-function-based indicator. The C-index of LFIS was 0.692 and time-dependent ROC curves at 6-, 12-, 18-, and 24- month of OS were 0.74, 0.714, 0.747, and 0.865, respectively. Based on the cutoff made by Xtile and efficacy and convenience of clinical application, the cohort could be further divided into four groups: LFIS score < 1 for Group 1, 1 < LFIS score ≤ 3 for Group 2, 3 < LFIS score ≤ 5 for Group 3, and LFIS score > 5 for Group 4. Kaplan–Meier survival analysis showed that patients with higher LFIS scores had worse OS compared to those with lower LFIS scores (Fig. 3B, C).

### The independent risk factors associated with overall survival

Univariate Cox regression analysis was utilized to identify the independent risk factors of OS among LFIS score and clinicopathological factors (Table 3). The correlation analysis (Supplementary Table 6) showed that LFIS score and clinicopathological factors had low correlations. All the variables had tolerance > 0.1 and VIF < 10, indicating there was no significant multicollinearity between these variables (Supplementary Table 7). Schoenfeld residuals analysis was performed to assess the proportional hazards assumption of these factors. As the results showed (Supplementary Table 8), both cycles of anti-PD1 and CRP with *P* < 0.05 were not satisfied for the proportional hazards assumption and were stratified in the multivariate Cox regression analysis. Whether receiving previous treatment was considered as an important clinical variable (*P* = 0.1) and also enrolled into multivariate Cox regression analysis. Finally, LFIS score, tumor number, and tumor diameter were regarded as independent prognostic factors (Table 3).

**Table 3:** Univariate and multivariate analyses of factors associated with overall survival survival in HCC patients

Variables		Univariate Cox regression			Multivariate Cox regression		
		HR	95%CI	*P*	HR	95%CIL	*P*
Gender	Female/male	1.308	0.840–2.037	0.235			
Age	< 60/ ≥ 60	0.937	0.679–1.294	0.693			
cycle of PD1	< 4/ ≥ 4	0.632	0.478–0.834	< 0.001			
Previous treatment	No/yes	1.263	0.956–1.667	0.100	1.148	0.788–1.673	0.473
BCLC	A–B/C	1.685	1.208–2.349	< 0.001	1.062	0.583–1.934	0.844
Extrahepatic metastasis	No/yes	1.643	1.242–2.173	< 0.001	1.416	0.97–2.065	0.071
Tumor number	0						
	1	1.676	0.763–3.685	0.199	1.550	0.673–3.573	0.303
	Multiple	2.621	1.227–5.602	0.013	2.277	1.015–5.107	0.046
Tumor diameter	< 10/ ≥ 10	2.156	1.631–2.850	< 0.001	1.682	1.2–2.358	0.003
Macrovascular invasion	No/yes	1.676	1.262–2.226	< 0.001	1.298	0.801–2.082	0.278
Combined therapy	No/yes	0.667	0.442–1.009	< 0.001			
CRP	< 10/ ≥ 10	1.497	1.131–1.981	0.001			
AFP	< 400/ ≥ 400	1.518	1.142–2.016	0.001	1.171	0.861–1.593	0.313
PNI		0.412	0.309–0.55	0.001	0.955	0.656–1.390	0.809
NLR		1.742	1.31–2.318	0.001	1.291	0.931–1.791	0.126
LFIS	1						
	2	2.501	1.536–4.072	< 0.001	2.147	1.244–3.704	< 0.001
	3	4.957	3.088–7.957	< 0.001	4.250	2.377–7.6	< 0.001
	4	7.514	4.586–12.312	< 0.001	4.752	2.499–9.036	< 0.001

HR, Hazard ratio; CI, confidence interval; LFIS, liver-function-indicators-based signature; BCLC, Barcelona Clinic Liver Cancer; AFP, alpha fetoprotein; CRP, C-reactive protein; PNI, prognostic nutritional index; NLR, neutrophil to lymphocyte rate

### The relationship between LFIS score and clinicopathological factors

Clinicopathological characteristics and treatment responses were compared between the LFIS score groups (Supplementary Table 9 and Supplementary Fig. 2). No difference was found among the four groups in age and agender. The four groups received similar previous treatments and combined therapy. HCC patients in LFIS Group 3 and Group 4 had worse liver functional reserve (higher Child–Pugh scores, ALT, AST, GGT, ALP, LDH, TBIL, and lower ALB levels) and had larger tumor diameters, more macrovascular invasion and higher AFP level. Moreover, patients in LFIS Group 3 and Group 4 had higher neutrophils, CRP, and NLR, but lower lymphocytes and PNI levels. The objective response rate (ORR) and disease control rate (DCR) in the LFIS Group 1 and Group 2 were significantly higher than those in the LFIS Group 3 and group 4. The median OS time in LFIS Group 1 and Group 2 were not reached. There were 23 deaths in LFIS Group1 and 55 deaths in LFIS Group2. Meanwhile, the 6-, 12 months OS rates in LFIS Group 1 were 93.97 and 82.76% and in Group 2 were 87.5 and 63.24%, respectively. Moreover, in LFIS Group 3 and Group 4, the median OS time was 12.2 months (10.9–14.7 months), and 9.08 months (6.7–12.9 months), respectively. The 6-, 12 months OS rates in LFIS Group 3 were 78.37 and 47.75% and in Group 4 were 60.56 and 30.99%, respectively.

## Discussion

ICI therapy and targeted therapy for advanced HCC have evolved remarkably over recent years. Previous studies showed that liver functional reserve was associated with anti-tumor treatment response and long-term prognosis of HCC patients with non-surgical treatments [30]. However, the predictors related to liver functional reserve with the best effective prognostic values for HCC patients who received anti-PD1 treatment remain unclear. In this retrospective study, we comprehensively investigated the prognostic values of 19 liver-function-based blood indicators for included HCC patients. We found that high Child–Pugh score, PAL, APAR, ALRI, and GLR levels were significantly correlated with poor prognosis. Based on five indicators, we further constructed a liver-function-indicators-based score. Patients were stratified into four groups with different liver function reserves, tumor response rate, and overall survival time. Moreover, we have further developed an online calculator for better application of our LFIS signature.

Previous studies have shown that patients who received anti-PD-1 treatment had a poor prognosis when they had high inflammatory scores [26, 31]. In this study, we also found that HCC patients in higher LFIS score groups were significantly associated with higher-level inflammatory biomarkers. Various studies suggested that baseline liver function has a significant impact on the efficacy of ICI therapy in advanced cancers [32–36]. For HCC, chronic liver disease could alter the immune microenvironment which has a significant influence on the anti-PD-1 therapy efficacy [37]. The Child–Pugh scoring system was widely used to assess liver functional reserve. However, in line with previous research [38, 39], most of the HCC patients in our study had Child–Pugh A liver functional reserve, and the Child–Pugh system had low predictive prognostic power and poor stratification ability. A study reported that a combination of ALP and GGT could accurately stratify the prognosis of HCC patients treated with PD-1 inhibitors with Child − Pugh grade A [40]. However, the reliability of this study was not strong enough because of the relatively small sample size and short follow-up time. In a larger population, our study developed an independent LFIS score based on the Child–Pugh score, PAL, APAR, ALRI, and GLR. This LFIS score could provide better survival and risk assessment for HCC patients with anti-PD-1 treatment.

APAR, consisting of ALP and ALB, was previously reported as a predictive biomarker in patients with pancreatic cancer and HCC [41, 42]. PAL score was significantly correlated with postoperative complications and survival outcomes of HCC patients with operation [18, 43]. ALRI and GLR, derived from liver function markers and inflammation makers, were also considered reliable indicators for prognostic prediction of HCC. Based on 434 HCC patients, our findings were consistent with the previous conclusion that confirmed PAL, APAR, ALRI, and GLR were independent prognostic factors for OS of HCC with anti-PD1-based therapy. As we all know, ALP and GGT are routine but important blood markers for the evaluation of liver function and could stratify these HCC patients with Child–Pugh grade A [40]. The elevated expression of ALP in HCC cells helped to enhance glycolysis and amino acid metabolism [44] and played an important role in promoting HCC cell proliferation [45, 46]. Studies have shown that elevated GGT is an independent future risk factor for cancers [47, 48]. Additionally, GGT as a glutathione-metabolism-related enzyme helps tumor cells maintain intracellular glutathione levels and may promote anti-tumor drug resistance [49]. Additionally, Serum ALB is another well-known and frequently used biomarker that reflects liver function and nutritional status of patients with various cancers. A multi-omics study pointed out that metabolic reprogramming in HCC suppressed the expression of ALB which could support the proliferation of HCC cells [50]. Low serum ALB level known as Hypoproteinemia also harms the sensitivity of anti-tumor treatments and leads to an imbalance in the tumor microenvironment. ALB and ICBs have similar pharmacokinetics and that is one of the reasons why ALB could be an independent predictor of treatment response and prognosis benefit of ICB therapy [51]. Moreover, maintaining the liver functional reserve during therapy could prolong the survival of HCC patients [52]. In this study, the newly built LFIS score has stronger stratification and prediction capabilities so that it could help recognize patients with poor liver functional reserve and provide closer surveillance and supportive approaches.

Inevitably, this study has limitations. Firstly, this was a retrospective and single-center study and the selection bias was objective. Secondly, the majority of patients in our cohort had HBV infection, which limited the extension of the applicability to other HCC patients with different liver disease backgrounds. It is necessary to validate the LFIS score in large HCC cohorts in other regions.

## Conclusion

In summary, our study developed a new LFIS score consisting of the Child–Pugh score, PAL, APAR, ALRI, and GLR. As an independent prognostic factor, a high LFIS score was associated with gloomy OS and PFS in HCC patients who received anti-PD-1 treatment. The LFIS score had excellent predictive and stratification ability compared with every single liver-function-based indicator. An online and easy-to-use calculator was further constructed for better application of this LFIS signature.

### Supplementary Information

Below is the link to the electronic supplementary material.

Supplementary file1 (PDF 816 KB)

## Data Availability

Source data are available from the corresponding author upon reasonable request.
