# Supplementary material for: A novel liver-function-indicators-based prognosis signature for patients with hepatocellular carcinoma treated with anti-programmed cell death-1 therapy
Source: Cancer Immunol Immunother. 2024 Jun 4;73(8):158. doi: 10.1007/s00262-024-03713-6 (PMC11150358; doi:10.1007/s00262-024-03713-6)
Supplement: Supplementary file 1 — Supplementary file1 (PDF 816 KB) [file 262_2024_3713_MOESM1_ESM.pdf]

Supplementary Material

Cancer Immunology, Immunotherapy (submitted in 2024) – Zehao Zheng. et al.

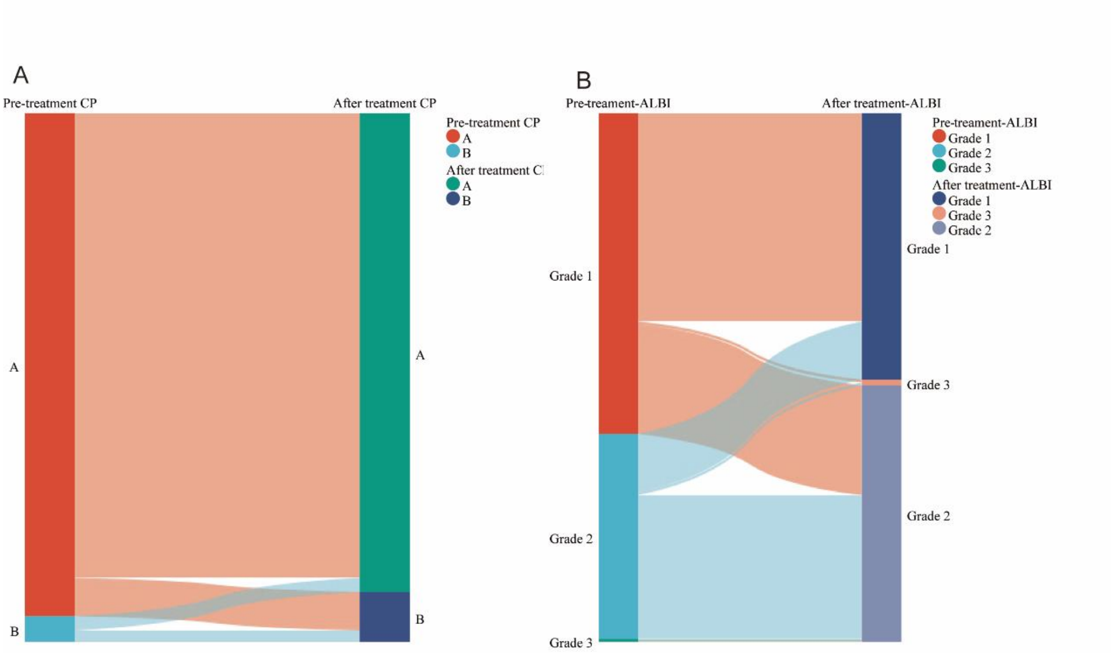

Supplementary Fig.1: (A–B) The Sankey Diagram of the liver function. (A) Child-Pugh grade, (B)ALBI grade.

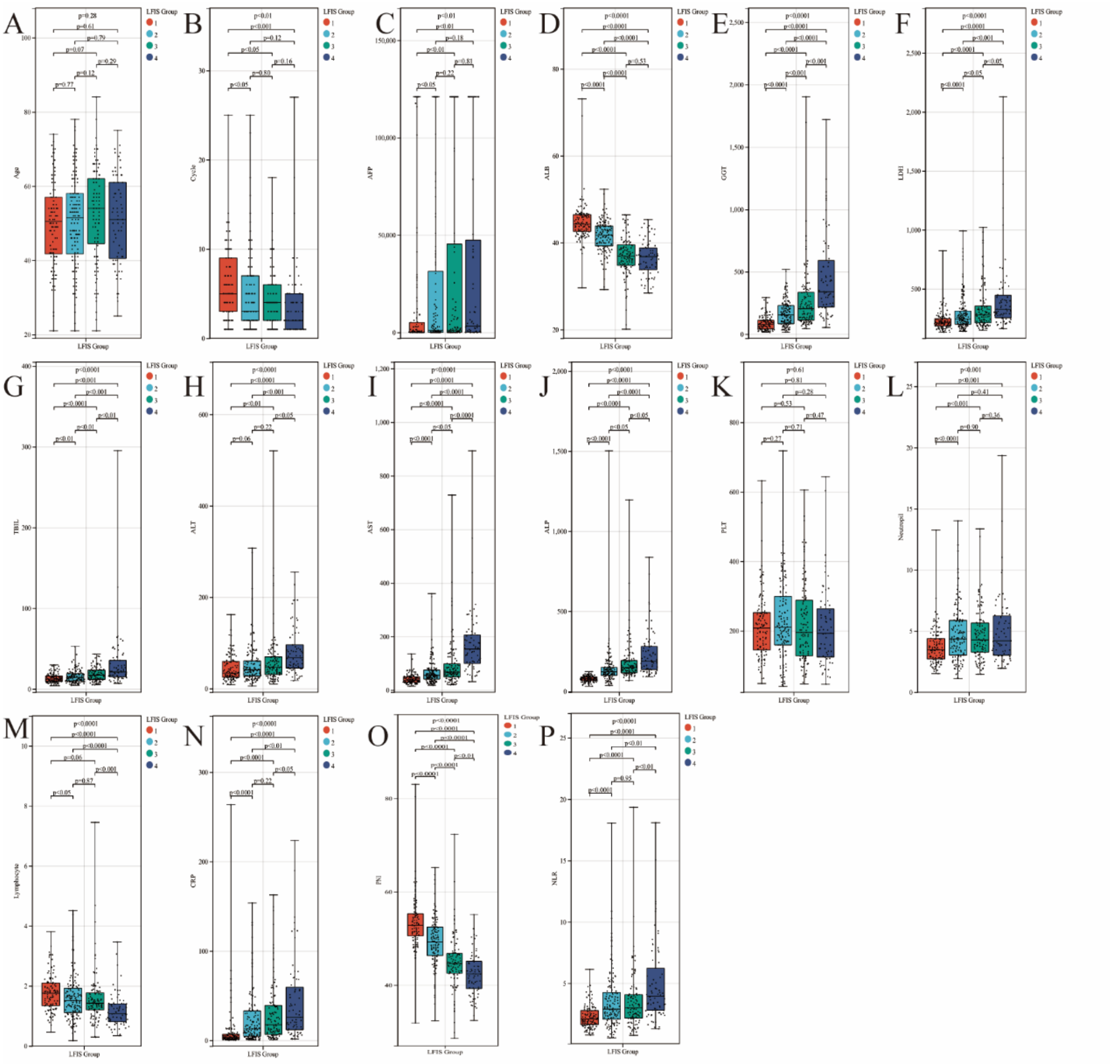

Supplementary Fig.2: (A–P) Clinicopathological characteristics of the patients grouped by LFIS score. (A) Age, (B)Cycle of anti-PD1 treatment, (C)AFP, (D) ALB, (E) GGT, (F) LDH, (G) TBIL, (H) ALT, (I) AST, (J)ALP, (K)PLT, (L)Neutrophil, (M)Lymphocyte, (N)CRP, (O)PNI, (P)NLR.

Supplementary Table 1. Category and dosage of PD-1 inhibitors

Category	Dose（mg）	No.(%)
Nivolumab	100	26(5.99)
Pembrolizumab	200	32(7.37)
Toripalimab	240	283(65.21)
Sintilimab	200	114(26.27)
Camrelizumab	200	25(5.76)

Abbreviations: No., Number.

Supplementary Table 2: The liver-function based indicators

Indicators	Abbreviations	Definition
Albumin-bilirubin scroe	ALBI	-0.085*albumin [g/L]-066 *log10(bilirubin [umol/L])
Platelet-albumin-bilirubin score	PALBI	2.02*log10 bilirubin [umol/L] −0.37∗ (log10 bilirubin [umol/L])^2-0.04 * albumin[g/L]-3.48 *log10 platelets count(10ˆ9/L) + 1.01 * (log10 platelet count(10ˆ9/L))^2
Platelet-albumin score	PAL	-0.777*albumin [g/dL]-0.575 *log10(platelet count [10ˆ4/μL])
neo-Glasgow prognostic score	neoGPS	neo-GPS 0：serum CRP level of < /=1.0 mg/dl and ALBI grade1.<br>neo-GPS 1：serum CRP level of >1.0 mg/dl or ALBI grade 2 or 3.<br>neo-GPS 2：serum CRP level of >1.0 mg/dl and ALBI grade 2 or 3.
Gamma-glutamyl transpeptidase to Platelet Ratio	GPR	γ-GT (U/L)/platelet count（10ˆ9/L）
Fibrosis-4	FIB4	age year *AST (U/L)/Platelet Count (1000/L)∗ ALT (U/L)
AST/ALT Ratio	AAR	AST (U/L)/ ALT (U/L)
aspartate-aminotransferase-to-neutrophil ratio index	APRI	AST/AST upper limit of normal (U/L)/ platelet count(10^9/L)
Alkaline phosphatase-to-albumin index	APAR	Alkaline phosphatase (U/L)/albumin(g/L)
Gamma-glutamyl transpeptidase-to-albumin	GAR	γ-GT (U/L)/albumin(g/L)
Gamma-glutamyl transpeptidase-to-lymphocyte ratio	GLR	γ-GT (U/L)/ lymphocytecount(10^9/L)
Aspartate aminotransferase-to-lymphocyte Ratio Index	ALRI	AST (U/L)/ lymphocytecount(10^9/L)
CRP-to-albumin ratio	CAR	CRP(mg/L)/albumin(g/L)

Supplementary Table 3: The correlations between tumor characteristics and liver function status

Characteristics	C-P A (N=403)	C-P B (N=31)	Total (N=434)	*P-value*	ALBI 1 (N=247)	ALBI 2-3 (N=187)	Total (N=434)	*P-value*
BCLC				0.17				0.59
A-B	118	5	123		73	50	123	
C	285	26	311		174	137	311	
Extrahepatic metastasis				0.6				0.43
No	247	17	264		146	118	264	
Yes	156	14	170		101	69	170	
Tumor number				0.66				
0	28	1	29		22	7	29	0.11
1	127	9	136		83	53	136	
multiple	248	21	269		142	127	269	
Tumor diameter				<0.05				<0.05
<10	246	9	255		166	89	255	
≥10	157	22	179		81	98	179	
Macrovascular invasion				0.05				<0.05
No	197	9	206		135	71	206	
Yes	206	22	228		112	116	228	

Abbreviations: BCLC, Barcelona Clinic Liver Cancer; C-P: Child-Pugh.

Supplementary Table 4: Univariate and Multivariate Cox Regression Analyses of the Liver-function based indicators for Overall Survival

Variables		Univariate Cox Regression Analyses			Stepwise Cox Regression Analysis		
		HR	95%CI	*P*	HR	95%CI	*P*
Child-Pugh	A/B	2.565	1.645-3.997	<0.001	1.884	1.151-3.083	0.012
GGT	< 281.4/≥281.4	2.476	1.856-3.304	<0.001			
LDH	< 259/≥259	2.149	1.627-2.838	<0.001			
AST	< 40/≥40	1.918	1.346-2.731	<0.001			
ALT	< 50/≥50	1.203	0.911-1.589	0.193			
ALB	< 35/≥35	0.527	0.3745-0.74	<0.001			
TBIL	< 20.5/≥20.5	2.007	1.501-2.683	<0.001			
PALBI	1						
	2	1.862	1.323-2.620	<0.001			
	3	3.068	2.13-4.419	<0.001			
PAL	1						
	2	2.193	1.646-2.922	<0.001	1.420	1.044-1.931	0.026
	3	1.990	1.005-3.941	0.048	0.950	0.449-2.010	0.894
neoGPS	0						
	1	1.904	1.329-2.728	<0.001			
	2	2.597	1.825-3.693	<0.001			
GPR	<0.69/≥0.69	2.109	1.579-2.818	<0.001			
FIB4	<88.15/≥88.15	2.365	1.699-3.291	<0.001			
AAR	<1.38/≥1.38	2.043	1.542-2.708	<0.001			
APRI	<2.46/≥2.46	2.289	1.55-3.38	<0.001			
APAR	<2.33/≥2.33	3.763	2.575-5.498	<0.001	2.678	1.787-4.013	<0.001
GAR	<5.73/≥5.73	2.683	2.026-3.552	<0.001			
GLR	<323.58/≥323.58	3.017	2.161-4.212	<0.001	1.787	1.246-2.564	<0.001
ALRI	<100.15/≥100.15	2.612	1.909-3.576	<0.001	1.799	1.281-2.526	<0.001
CAR	< 0.13/≥0.13	2.104	1.523-2.906	<0.001			

Abbreviations: HR, Hazard ratio; CI, confidence interval; ALB, albumin; ALT, alanine aminotransferase; AST, aspartate aminotransferase; GGT, gamma-glutamyl transpeptidase; TBIL, total bilirubin; PALBI, platelet-albumin-bilirubin index; PAL, platelet-albumin index; neoGPS, neo-Glasgow prognostic score; APAR, alkaline phosphatase-to-albumin index; GAR, gamma-glutamyl transpeptidase-to- albumin ratio; GPR, gamma-glutamyl transpeptidase-to-platelet ratio;FIB-4, fibrosis-4; AAR, AST/ALT ratio; ALRI, aspartate aminotransferase-to-lymphocyte ratio index; GLR, gamma-glutamyl transpeptidase-to-lymphocyte ratio CAR, C-reactive protein to albumin ratio.

Supplementary Table 5: The details of formula

Formula	the value of indicators	βi(coefficient)	(βi(coefficient) * the value of an indicator
Exp[Σ(βi(coefficient) * the value of an indicator]	Child-Pugh		
	0(A)	0	0
	1(B)	0.63319	0.63319
	PAL		
	0 (grade1)	0	0
	1 (grade2)	0.35054	0.35054
	2 (grade3)	-0.05118	-0.10236
	APAR		
	0(<2.33)	0	0
	1（⩾2.33)	0.98514	0.98514
	GLR		
	0(<323.58)	0	0
	1（⩾323.58)	0.58049	0.58049
	ALRI		
	0(<100.15)	0	0
	1（⩾100.15)	0.58714	0.58714

Abbreviations: PAL, platelet-albumin index; APAR, alkaline phosphatase-to-albumin index; ALRI, aspartate aminotransferase-to-lymphocyte ratio index; GLR, gamma-glutamyl transpeptidase-to-lymphocyte ratio.

Supplementary Table 6: The correlation analysis of variables

		LFIS group	PNI	NLR	CRP	Previous treatment	BCLC	Extrahepatic Metastasis	Tumor number	Tumor diameter	AFP	Cycle of PD1	Combined therapy	Macrovascular invasion
LFIS group	R	1.000	-.577**	.286**	.365**	0.001	.096*	-0.001	.117**	.295**	.121**	-.131**	-0.079	.225**
	P-value		0.000	0.000	0.000	0.988	0.030	0.980	0.006	0.000	0.001	0.003	0.063	0.000
	N	434	434	434	434	434	434	434	434	434	434	434	434	434
PNI	R	-.577**	1.000	.286**	-.291**	-0.006	-0.021	0.048	-0.065	-.211**	-0.053	.160**	0.043	-.144**
	P-value	0.000		0.000	0.000	0.899	0.658	0.322	0.162	0.000	0.179	0.001	0.349	0.003
	N	434	434	434	434	434	434	434	434	434	434	434	434	434
NLR	R	.286**	-.323**	1.000	.275**	-0.095	0.012	-0.031	0.065	.121*	-0.015	-0.163	-0.003	0.07433579
	P-value	0	0.000		0.000	0.049	0.800	0.519	0.162	0.012	0.699	0.001	0.951	0.122
	N	434.000	434	434	434	434	434	434	434	434	434	434	434	434
CRP	R	.365**	-.291**	.275**	1.000	-.154**	0.056	0.002	.112*	.324**	.106**	-.155**	0.038	.151**
	P-value	0.000	0.000	0.000		0.001	0.243	0.960	0.016	0.000	0.008	0.001	0.413	0.002
	N	434	434	434	434	434	434	434	434	434	434	434	434	434
Previous treatment	R	0.001	-0.006	-0.095	-.154**	1.000	0.083	.265**	-0.036	-.294**	-.112**	0.048	-.452**	-.170**
	P-value	0.988	0.899	0.049	0.001		0.086	0.000	0.444	0.000	0.005	0.315	0.000	0.000

	N	434	434	434	434	434	434	434	434	434	434	434	434	434
BCLC	R	.096*	0.012	0.012	0.056	0.083	1.000	.421**	-0.091	-0.013	.114**	0.044	-.200**	.651**
	P-value	0.030	0.800	0.800	0.243	0.086		0.000	0.051	0.784	0.004	0.357	0.000	0.000
	N	434	434	434	434	434	434	434	434	434	434	434	434	434
Extrahepatic Metastasis	R	-0.001	0.048	-0.031	0.002	.265**	.421**	1.000	-0.046	-0.049	-0.015	.107*	-.370**	-.107*
	P-value	0.980	0.322	0.519	0.960	0.000	0.000		0.325	0.307	0.702	0.025	0.000	0.026
	N	434	434	434	434	434	434	434	434	434	434	434	434	434
Tumor number	R	.117**	-0.065	0.065	.112*	-0.036	-0.091	-0.046	1.000	0.063	.078*	-0.068	-0.017	0.015
	P-value	0.006	0.162	0.162	0.016	0.444	0.051	0.325		0.179	0.043	0.146	0.703	0.742
	N	434	434	434	434	434	434	434	434	434	434	434	434	434
Tumor diameter	R	.295**	-.211**	.121*	.324**	-.294**	-0.013	-0.049	0.063	1.000	.238**	-0.071	.108*	.178**
	P-value	0.000	0.000	0.012	0.000	0.000	0.784	0.307	0.179		0.000	0.142	0.020	0.000
	N	434	434	434	434	434	434	434	434	434	434	434	434	434
AFP	R	.121**	-0.053	-0.015	.106**	-.112**	.114**	-0.015	.078*	.238**	1.000	0.012	0.012	.152**
	P-value	0.001	0.179	0.699	0.008	0.005	0.004	0.702	0.043	0.000		0.762	0.757	0.000
	N	434	434	434	434	434	434	434	434	434	434	434	434	434
Cycle of PD1	R	-.131**	.160**	-0.163	-.155**	0.048	0.044	.107*	-0.068	-0.071	0.012	1.000	0.036	-0.036

	P-value	0.003	0.001	0.001	0.001	0.315	0.357	0.025	0.146	0.142	0.762		0.438	0.449
	N	434	434	434	434	434	434	434	434	434	434	434	434	434
Combined therapy	R	-0.079	0.043	-0.003	0.038	-.452**	-.200**	-.370**	-0.017	.108*	0.012	0.036	1.000	0.018
	P-value	0.063	0.349	0.951	0.413	0.000	0.000	0.000	0.703	0.020	0.757	0.438		0.704
	N	434	434	434	434	434	434	434	434	434	434	434	434	434
Macrovascular invasion	R	.225**	0.074	-.213**	.151**	-.170**	.651**	-.107*	0.015	.178**	.152**	-0.036	0.018	1.000
	P-value	0.000	0.122	0.000	0.002	0.000	0.000	0.026	0.742	0.000	0.000	0.449	0.704	
	N	434	434	434	434	434	434	434	434	434	434	434	434	434

Abbreviations: LFIS, liver-function-indicators-based signature; BCLC, Barcelona Clinic Liver Cancer; AFP, alpha fetoprotein; CRP, C-reactive protein; PNI, prognostic nutritional index; NLR, neutrophil to lymphocyte rate. *=P<0.05; **=P<0.01.

Supplementary Table 7: The Tolerance and VIF of the Variables

Variables	Tolerance	VIF
LFIS group	0.509	1.963
BCLC	0.303	3.296
Previous treatment	0.686	1.458
Extrahepatic metastasis	0.513	1.949
Tumor number	0.929	1.076
Tumor diameter	0.691	1.448
Macrovascular invasion	0.346	2.887
Combined therapy	0.718	1.392
Cycle of PD1	0.931	1.075
CRP	0.756	1.322
AFP	0.843	1.186
PNI	0.585	1.708
NLR	0.836	1.196

Abbreviations: LFIS, liver-function-indicators-based signature; BCLC, Barcelona Clinic Liver Cancer; AFP, alpha fetoprotein; CRP, C-reactive protein; PNI, prognostic nutritional index; NLR, neutrophil to lymphocyte rate.

Supplementary Table 8: The results of Schoenfeld residuals analysis

Variables	***P-value***
LFIS group	0.33
Previous treatment	0.48
Tumor number	0.81
Tumor diameter	0.7
Extrahepatic metastasis	0.81
Macrovascular invasion	0.78
BCLC	0.75
AFP	0.8
Cycle of anti-PD-1	0.01
Combined therapy	0.02
CRP	0.01
NLR	0.16
PNI	0.6
GLOBAL	0.05

Abbreviations: LFIS, liver-function-indicators-based signature; BCLC, Barcelona Clinic Liver Cancer; AFP, alpha fetoprotein; CRP, C-reactive protein; PNI, prognostic nutritional index; NLR, neutrophil to lymphocyte rate.

Supplementary Table 9: Baseline Characteristics of the Patients Grouped by LFIS Score

Variables		LFIS Group				*P*-value
		1	2	3	4	
Gender	Female	19 (0.32)	19 (0.32)	15 (0.26)	6 (0.10)	0.497
	Male	97 (0.26)	117 (0.31)	96 (0.26)	65 (0.17)	
Previous treatment	No	67 (0.27)	76 (0.31)	63 (0.25)	41 (0.17)	0.990
	Yes	49 (0.26)	60 (0.32)	48 (0.26)	30 (0.16)	
BCLC	A-B	45 (0.37)	31 (0.25)	31 (0.25)	16 (0.13)	0.023
	C	71 (0.23)	105 (0.34)	80 (0.25)	55 (0.18)	
Child-Pugh	A	116 (0.29)	134 (0.33)	105 (0.26)	48 (0.12)	<0.001
	B	0 (0)	2 (0.06)	6 (0.19)	23 (0.75)	
Extrahepatic Metastasis	No	70 (0.26)	82 (0.31)	71 (0.27)	41 (0.16)	0.857
	Yes	46 (0.27)	54 (0.32)	40 (0.24)	30 (0.17)	
Tumor Number	0	12 (0.41)	12 (0.41)	3 (0.1)	2 (0.07)	0.098
	1	41 (0.30)	42 (0.31)	33 (0.24)	20 (0.15)	
	Multiple	63 (0.23)	82 (0.31)	75 (0.28)	49 (0.18)	
Tumor Diameter	0-10cm	93 (0.37)	84 (0.33)	54 (0.21)	24 (0.09)	<0.001
	> 10cm	23 (0.13)	52 (0.29)	57 (0.32)	47 (0.26)	
Macrovascular invasion	No	78 (0.38)	63 (0.30)	41 (0.20)	24 (0.12)	<0.001
	Yes	38 (0.17)	73 (0.32)	70 (0.31)	47 (0.20)	
Hepatitis	No	16 (0.26)	23 (0.37)	14 (0.23)	9 (0.14)	0.755
	Yes	100 (0.27)	113 (0.30)	97 (0.26)	62 (0.17)	
Combined therapy	No	11 (0.23)	18 (0.38)	12 (0.25)	7 (0.15)	0.787
	Yes	105 (0.27)	118 (0.31)	99 (0.26)	64 (0.17)	
BR	NA	9 (0.13)	18 (0.25)	18 (0.25)	26 (0.37)	<0.001
	CR	6 (0.27)	7 (0.32)	6 (0.27)	3 (0.14)	
	PR	41 (0.38)	32 (0.3)	24 (0.22)	10 (0.09)	
	SD	47 (0.29)	47 (0.29)	51 (0.31)	17 (0.1)	
	PD	13 (0.18)	32 (0.44)	12 (0.17)	15 (0.21)	
DCR	Non-response	13 (0.18)	32 (0.44)	12 (0.17)	15 (0.21)	<0.001
	Response	94 (0.32)	86 (0.3)	81 (0.28)	30 (0.1)	
	NA	9 (0.13)	18 (0.25)	18 (0.25)	26 (0.37)	

Abbreviations: BCLC, Barcelona Clinic Liver Cancer; BR, best response; NA, not assessed; CR, complete response; PR, partial response; SD, stable disease; PD, progressive disease; DCR, disease control rate.
